# Proteomics identifies *Bacillus cereus* EntD as a pivotal protein for the production of numerous virulence factors

**DOI:** 10.3389/fmicb.2015.01004

**Published:** 2015-10-07

**Authors:** Hélène Omer, Béatrice Alpha-Bazin, Jean-Luc Brunet, Jean Armengaud, Catherine Duport

**Affiliations:** ^1^Université d'Avignon et des Pays de Vaucluse, UMR408 Sécurité et Qualité des Produits d'Origine VégétaleAvignon, France; ^2^INRA, UMR408 Sécurité et Qualité des Produits d'Origine VégétaleAvignon, France; ^3^CEA-Marcoule, DSV/IBITEC-S/SPI/Li2D, Laboratory “Innovative technologies for Detection and Diagnostic”Bagnols-sur-Cèze, France; ^4^INRA, UR406 Abeilles et EnvironnementAvignon, France

**Keywords:** comparative proteomics, cellular proteome, exoproteome, *Bacillus cereus*, metabolism, virulence

## Abstract

*Bacillus cereus* is a Gram-positive pathogen that causes a wide variety of diseases in humans. It secretes into the extracellular milieu proteins that may contribute directly or indirectly to its virulence. EntD is a novel exoprotein identified by proteogenomics of *B. cereus* ATCC 14579. We constructed a Δ*entD* mutant and analyzed the impact of *entD* disruption on the cellular proteome and exoproteome isolated from early, late, and stationary-phase cultures. We identified 308 and 79 proteins regulated by EntD in the cellular proteome and the exoproteome, respectively. The contribution of these proteins to important virulence-associated functions, including central metabolism, cell structure, antioxidative ability, cell motility, and toxin production, are presented. The proteomic data were correlated with the growth defect, cell morphology change, reduced motility, and reduced cytotoxicity of the Δ*entD* mutant strain. We conclude that EntD is an important player in *B. cereus* virulence. The function of EntD and the putative EntD-dependent regulatory network are discussed. To our knowledge, this study is the first characterization of an Ent family protein in a species of the *B. cereus* group.

## Introduction

Microbial pathogens are involved in a wide range of severe, and sometimes fatal, human diseases, including nosocomial infections, foodborne infections and toxic shock syndrome. The pathogenesis of the majority of bacterial diseases is a multifactorial process. Five common, crucial steps may be listed: (i) resistance to environmental stresses during infection, (ii) adhesion to the host cell, (iii) invasion, (iv) damage to host tissues, and (v) subversion of the host immune response (Finlay and Falkow, 1997). Completion of each stage is dependent on orchestrated activities of specific exoproteins. Exoproteins, which play a pivotal role in the adaptability of the pathogen to the specificities of the host's intracellular environment and promote efficient infection, are recognized as virulence factors (Wilson et al., 2002).

*Bacillus cereus* is a Gram-positive, motile, rod-shaped bacterium closely related to *Bacillus anthracis* and *Bacillus thuringiensis*. *B. anthracis* is a notorious pathogen that causes anthrax in human and animals (Goel, 2015). *B. thuringiensis* is an insect pathogen used in crop protection (Drobniewski, 1993; Pardo-López et al., 2013). *B. cereus* is primarily associated with foodborne gastrointestinal (GI) infections but it is also responsible for systemic diseases and nosocomial infections (Kotiranta et al., 2000; Bottone, 2010; Ramarao and Sanchis, 2013). *B. cereus* is also highly associated with endophthalmitis (Callegan et al., 2007). The pathogenicity of *B. cereus* in endophthalmitis is associated not only with the production of virulence factors but also with the inflammogenicity of the cell wall and the motility of the bacterium within the eye (Callegan et al., 2005; Parkunan et al., 2014). *B. cereus* uses peritrichous flagella as motility appendages (Salvetti et al., 2007). Flagellum assembly takes place in the context of the whole cell and is mediated by the flagellar export apparatus, which is homologous to a type III secretion system (Guttenplan et al., 2013). The contribution of this flagellar apparatus to *B. cereus* virulence has been documented by several studies (Ghelardi et al., 2007; Salvetti et al., 2007; Senesi et al., 2010). Flagella are also important for biofilm formation (Houry et al., 2010). Bacteria inside biofilms are protected from the host immune system, antimicrobials, and other external insults (Flemming and Wingender, 2010). Biofilm formation in *B. cereus* involves the main virulence regulator PlcR; this points toward the inevitable connection of virulence factor production with biofilm formation in this bacterium (Hsueh et al., 2006).

Previous shotgun proteomic studies provided evidence that *B. cereus*, like other pathogens, can deploy a large arsenal of virulence factors to promote infection (Clair et al., 2010, 2013; Laouami et al., 2014; Madeira et al., 2015). Some of these virulence factors are abundant components of the *B. cereus* exoproteome (Madeira et al., 2015). Among them are the three most extensively studied diarrheal enterotoxins: hemolysin BL (Hbl), nonhemolytic enterotoxin (Nhe), and cytotoxin K (CytK). Hbl is composed of three distinct protein components, L2, L1, and B, which are all required to obtain full enterotoxigenic activity. Nhe is also a three-component enterotoxin. The three components, NheA, NheB, and NheC, differ from those of Hbl. Unlike Hbl and Nhe, CytK is a single component protein (Beecher et al., 1995; Stenfors Arnesen et al., 2008). Hbl, Nhe, and CytK have shown strong disruptive effects on epithelial cells and all the corresponding genes belong to the PlcR virulence regulon (Gohar et al., 2008). In addition, the *B. cereus* exoproteome contains numerous PlcR-independent virulence factors. Some of these have been shown to play a role in *B. cereus* pathogenicity, such as InhA1 and HlyII (Ramarao and Lereclus, 2005; Andreeva et al., 2006; Guinebretiere et al., 2010). Many are still uncharacterized, such as the proteins EntA, EntB, and EntC, which are major contributors to the *B. cereus* exoproteome (Clair et al., 2010). The abundance level of virulence factors detected in the *B. cereus* exoproteome is regulated in response to challenging conditions. This is partly achieved through the regulation of specific genes for each particular growth condition but also by the activation of global metabolic regulatory pathways such as those involving the repressor of branched amino acids CodY (Lindbäck et al., 2012), and the redox regulators ResDE, Fnr, Rex, and OhrRA (Duport et al., 2006; Zigha et al., 2007; Esbelin et al., 2008, 2009; Messaoudi et al., 2010). All of these regulators contribute to the maintenance of intracellular homeostasis during growth.

Despite the identification and characterization of several virulence factors, the pathogenicity of *B. cereus* is still poorly understood and needs further investigation. The aim of this study was to evaluate the role of EntD, an exoprotein identified by proteogenomics of *B. cereus* ATCC 14579 (Ivanova et al., 2003), in the virulence of *B. cereus* ATCC 14579 by in-depth characterization of an *entD* knockout mutant. We used proteomics to investigate the profiles and functions of cellular and extracellular proteins controlled by EntD. Proteomic data were correlated with cell structure, metabolic, and phenotypic changes. The results indicate that EntD is a key extracellular virulence-associated factor, which regulates bacterial processes such as, motility, and toxin production, through an as-yet unidentified signaling pathway.

## Materials and methods

### Bacterial strains and growth conditions

Wild-type *B. cereus* ATCC 14579 without its pBClin15 plasmid (Ivanova et al., 2003) and an Δ*entD* mutant were grown in MOD medium supplemented with 30 mM glucose as the carbon source, as previously described (Rosenfeld et al., 2005). Non-controlled aerobic batch cultures were performed in 3 L flasks containing 500 mL culture medium. Flasks were incubated with shaking (200 rpm) at 37°C. The inoculum was a sample of an overnight culture harvested by centrifugation, washed and diluted in fresh medium to obtain an initial optical density at 600 nm of 0.02. Anaerobic cultures were performed in 20 mL Hungate tubes equipped with open-top caps and rubber septa. Each vial was filled with 10 mL culture medium. Oxygen-free N_2_ gas was vigorously flushed through a needle into the culture medium for 3 min after inoculation. Tubes were incubated with stirring (200 rpm) at 37°C. In control experiments (cell-free systems), resazurin bleaching was monitored to ensure that the conditions were fully anaerobic. *B. cereus* growth was monitored spectrophotometrically at 600 nm. The specific growth rate (μ) was determined using the modified Gompertz equation (Zwietering et al., 1990, 1992). Samples (50 mL) were harvested at early exponential growth phase (EE), late exponential growth phase (LE), and stationary phase (S) for proteome analysis. Cells and culture supernatants were separated by centrifugation at 10,000 × *g* for 10 min at 4°C. Cell pellets were immediately stored at -80°C until analysis. Culture supernatants were first filtered through a low-adsorption cellulose acetate membrane filter with 45 μm pore size and then through a 20-μm-pore-size filter. Filtered fractions (40 mL) were stored at −80°C until analysis.

### Construction of Δ*entD* mutant cells and complementation

A *Bam*HI-*EcoR*I DNA fragment of 980 bp encompassing the *BC_3716* locus was amplified by PCR using chromosomal DNA as the template and the primer pair 5′-ggatccAAATTCTAAAAATCTGTTGCTATAATG-3′ plus 5′-gaattcTTCGCCCCCAGCTATTAGGACTA-3′. The amplified DNA fragment was cloned into pCRXL-TOPO (Invitrogen). The resulting pCRXLentD plasmid was digested with *Psi*I. A 1.5 kb *SmaI* fragment containing the spectinomycin resistance expression cassette spc (Murphy, 1985) was purified from pDIA (Laouami et al., 2011) and ligated into *Psi*I-digested pCRXLentD. The resulting plasmid, pCRXL-*entD*Δspc, was digested with *EcoR*I plus *BamH*I. The *entD*Δspc-*entD* fragment was subsequently inserted between the corresponding pMAD sites. The resulting plasmid was introduced into *B. cereus* strains by electroporation. The *entD* gene was deleted by a double crossover event (Arnaud et al., 2004). Chromosomal allele exchanges were confirmed by PCR with oligonucleotide primers located upstream and downstream of the DNA regions used for allelic exchange. To complement the *entD* gene *in trans*, the 1429 bp *entD* locus was first amplified using the primer pair 5′- gaattcTTGGTAAAAGATGTAACGAATTGTG-3′and 5′- agatctCGCCCTAAATTGTTTACTACGG-3′ and then cloned into pCRXL-TOPO. The PCR fragment was cut with Bam*HI* and *Bgl*II and ligated to similarly digested pHT304 (Arantes and Lereclus, 1991). The integrity of the insert in the recombinant vector was verified by sequencing and the vector was then used to transform the *B. cereus* mutant strain.

### Protein sample preparation for shotgun proteomics

Cell pellets were thawed on ice and extracted as previously described (Clair et al., 2012). Fractions of 40 mL *B. cereus* supernatants were thawed on ice and precipitated using the deoxycholate/trichloroacetic acid method as previously described (Clair et al., 2010; Madeira et al., 2015). Intracellular proteins from the 6 samples (biological triplicates for the wild-type and the mutant strains) and extracellular proteins from the 18 samples (biological triplicates from the 3 time-conditions for the wild-type and mutant strains) were resolved on NuPAGE Novex 4–12% Bis-Tris gels (Invitrogen) with a short migration of 5 min at 200 V using MES supplemented with antioxidant solution as running buffer and as previously described (Hartmann and Armengaud, 2014; Hartmann et al., 2014). Gels were stained with Simply Blue Safe Stain, a ready-to-use Coomassie G-250 stain from Invitrogen. After destaining, the proteins of each gel lane were cut as a single band, which was then further divided into 2 fractions, each corresponding to a 3 × 4 mm^2^ polyacrylamide band. The resulting polyacrylamide gel pieces were processed for further destaining, iodoacetamide treatment, and in-gel proteolysis with trypsin (Roche) in the presence of ProteaseMax additive (Promega), as previously described (Clair et al., 2010). The two digests obtained from the same sample were pooled as a single peptide mixture. Exponential phase samples were injected without being diluted, due to their lower protein content, while the samples collected at LE and S growth phases were diluted 1:20 in 0.1% trifluoroacetic acid prior to nanoLC-MS/MS analysis.

### NanoLC-MS/MS analysis

Technical duplicates were carried out for the 6 intracellular samples and the 18 extracellular samples, resulting in 12 and 36 samples, respectively. NanoLC-MS/MS analysis was performed for each technical replicate on an LTQ-Orbitrap XL hybrid mass spectrometer (ThermoFisher) coupled to an UltiMate 3000 nRSLC system (Dionex-ThermoFisher). This system was operated as described previously (Clair et al., 2012). Peptide mixtures (5 μL) were loaded and desalted on-line on a reverse-phase precolumn (Acclaim PepMap100 C18, 5 μm bead size, 100 Å pore size, 300 μm i.d. × 5 mm, Dionex-ThermoFisher). Peptides were then resolved on a Dionex nanoscale C_18_ PepMap 100™ C_18_ capillary column (3 μm bead size, 100-Å pore size, 75 μm i.d. × 15 cm) at a flow rate of 0.3 μL/min with a gradient of CH_3_CN, 0.1% formic acid prior to injection into the ion trap mass spectrometer. Peptides were separated using a 90 min gradient from 4 to 40% solvent B (0.1% HCOOH, 100% CH_3_CN). Solvent A was 0.1% HCOOH, 100% H_2_O. Full-scan mass spectra were measured from *m*/*z* 300 to 1800 with the LTQ-Orbitrap XL mass spectrometer in data-dependent mode using the TOP3 strategy. In brief, a scan cycle was initiated with a full scan of high mass accuracy in the Orbitrap followed by MS/MS scans in the linear ion trap on the three most abundant precursor ions with 60 s dynamic exclusion of previously selected ions. For cell pellet extracts the column used was 50 cm long and was operated with a 180 min gradient.

### Assignment of MS/MS spectra to peptide sequences and protein validation

Peak lists were generated with the MASCOT DAEMON software (version 2.3.2) from Matrix Science using the extract_msn.exe data import filter from the Xcalibur FT package (version 2.0.7) proposed by ThermoFisher. Data import filter options were set as follows: minimum mass, 400 m/z; maximum mass, 5000 m/z; grouping tolerance, 0; intermediate scans, 0; and threshold, 1000. Using the MASCOT search engine (version 2.3.02) from Matrix Science, we searched all MS/MS spectra against an in-house polypeptide sequence database (BCRUS_09) containing the sequences of all annotated proteins encoded by the *B. cereus* ATCC 14579 chromosome (NC_004722) and the plasmid pBClin15 (NC_004721) and proteins identified by a proteogenomic study (Madeira et al., 2015 and unpublished results). This database comprises 5299 polypeptide sequences, totaling 1,464,675 amino acids. Searches for tryptic peptides were performed with the following parameters: full trypsin specificity, mass tolerance of 5 ppm on the parent ion and 0.6 Da on the MS/MS, static modifications of carboxyamidomethylated Cys (+57.0215), and dynamic modifications of oxidized Met (+15.9949). The maximum number of missed cleavages was set at 2. All peptide matches with a peptide score below a *p*-value of 0.05 were filtered by the IRMa 1.28.0 parser (Dupierris et al., 2009). A protein was considered validated when at least two different peptides were detected in the same sample. The false-positive rate for protein identification was estimated using the appropriate decoy database as below 0.1% with these parameters. The mass spectrometry proteomics data have been deposited in the ProteomeXchange Consortium (http://proteomecentral.proteomexchange.org) via the PRIDE partner repository (http://www.ebi.ac.uk/pride) with the dataset identifiers PXD001482 and PXD001483.

### Spectral count-based protein quantification and statistical analysis

The number of MS/MS spectra per protein was determined in the 3 different nanoLC-MS/MS biological replicates for each condition. Proteins were further considered for comparison only if peptides were seen in at least 3 of the nanoLC-MS/MS experiments for a specific condition. Outlier data points were not removed. The data from each growth condition were compared by a T-Fold method using PatternLab software 2.0.0.13 (Carvalho et al., 2008). The fold change and *p*-value cut-offs were set at 1.5 and 0.01, respectively. The false discovery rate with these settings was below 15%.

### Hydrogen peroxide killing assays

B. cereus ATCC 14579 and Δ*entD* mutant were grown to mid-log phase (OD_600_~ 0.3) in MOD medium supplemented with 30 mM glucose. The cells were then centrifuged and resuspended in an equal volume of phosphate-buffered saline solution (PBS). Hydrogen peroxide challenge assays were assessed by exposing samples to 0.5 and 1 mM H_2_O_2_, respectively. Aliquots (100 μL) of the samples were diluted in H_2_O, appropriate dilutions of the culture were plated onto LB agar, and after overnight incubation at 37°C the colony forming units (CFUs) were counted. All the experiments were performed at least in triplicate, and at least 2 technical replicates from each dilution step were carried out to determine the number of CFUs.

### Motility, biofilm, and autolysis assays

The swimming and swarming abilities of *B. cereus* ATCC 14579 and Δ*entD* mutant strains were determined on TrB (Tryptone, 10 g/L; NaCl, 5 g/L) plates containing 2% and 0.2% (w/v) Bacto-agar (Difco), respectively (Salvetti et al., 2007). The cells were grown to mid-log phase and the OD_600_ was adjusted to 0.5. Five microliters of cell suspensions were spotted onto the center of TrB plates, and growth halo diameters were measured after 1 to 15 days of incubation at 37°C.

Biofilm formation of *B. cereus* ATCC 14579 and Δ*entD* mutant strains were determined as follows. Cells were grown overnight at 37°C in BHI medium (Biokar Diagnostics), washed with BHI medium and diluted to an OD_600_ of 1. Two hundred microliters were added to the wells of 96-well plates and incubated for 24 h at 37°C without agitation. Biofilms were stained with 200 μL 1% (w/v) crystal violet for 20 min and washed 3 times with PBS. Crystal violet bound to biofilm was solubilized using 200 μL 100% ethanol. Biofilms were quantified by measuring the optical density due to crystal violet at 590 nm. Assays were repeated 10 times.

The autolytic rates of *B. cereus* ATCC 14579 and Δ*entD* mutant strains were determined from cells grown in MOD medium supplemented with 30 mM glucose and harvested at mid-log phase (OD_600_ ~ 0.3). The cells were washed with PBS, resuspended in the same buffer to an OD_600_ ~ 0.5 and incubated at 37°C. The autolytic rate was expressed as a percentage decrease of the OD_600_ after 72 h (Raddadi et al., 2005). These experiments were performed in triplicate.

Statistical difference between the wild-type and *entD* strains was evaluated by Student's *t*-test.

### Electron microscopy

*B. cereus* ATCC 14579 and Δ*entD* mutant strains were grown to mid-log phase (OD_600_~0.3) in MOD medium supplemented with 30 mM glucose. Ultrathin sections of bacteria were prepared as previously described (Pandiani et al., 2010). Observations at high magnification (up to 35,000x) were performed under a transmission electron microscope (TEM; FEI-Philips CM10; Philips, Eindhoven, The Netherlands). Negative staining was used to visualize flagella at 7000x magnification.

### Cytotoxicity toward Caco-2 cells

Human colon adenocarcinoma Caco-2 cells were cultured in high-glucose Dulbecco's modified Eagle's medium (DMEM, Ozyme) supplemented with 1% nonessential amino acids and 10% fetal calf serum. Cells were incubated at 37°C under an atmosphere of 5% CO_2_ and 95% relative humidity. The culture medium was replaced three times a week. Passage was performed at 80% confluence. All studies were performed between passages 44 and 56. For cytotoxicity tests, cells were seeded in 96-well culture plates in 100 μL culture medium at a density of 1.5 × 10^4^ cells/well. Ten to fifteen days after seeding, cells formed a dome epithelium and were exposed to bacterial exoproteins. Exoprotein samples were prepared from *B. cereus* ATCC 14579 and Δ*entD* supernatants harvested at EE, LE and S growth phases (Figure S1) and diluted in DMEM to reach a final protein amount of either 10 or 100 μg in 20 μL. Cytotoxicity was assessed by measuring the mitochondrial activity using the permeant tetrazolium salt 3-(4,5-dimethylthiazol-2yl)-2,5di-phenyltetrazolium bromide (MTT) as previously described (Vidau et al., 2009). Briefly, after 21, 45 and 69 h exposure to bacterial exoproteins, 50 μL MTT (0.5 mM in PBS) were added and cells were incubated for 3 h at 37°C. The cells were then washed three times with PBS and formazan crystals were dissolved with 200 μL DMSO. The absorbance at 550 nm was recorded using 100 μL of the solution. Each experiment was repeated at least three times in quadruplicate.

### Mapping transcriptional start site and real-time RT-PCR analyses

Total RNA was isolated from cell pellets as previously described (Brillard et al., 2010). The 5′-end of *entD* mRNA was mapped as previously described (Clair et al., 2010). The *entD* specific primers SP1, SP2 and SP3 were: 5′-TGAACATATCCAACTTTT-3′, 5′-GCACGAACATTTA ATACGCCC-3′and 5′-TGTTTCCGC TTTAGCAGAACC-3′, respectively. Real-time RT-PCR was performed using the iScript™ One-Step RT-PCR Kit with SYBR® Green following the manufacturer's protocol (Biorad). The reactions were run using the MiniOpticon Real-Time PCR (BioRad). The *entD*-specific primer pair used in this study was: 5′-CGAAAACAAAGCAAATGGAGGC-3′ and 5′-TCCCACTGAACACTTTACCTACA-3′. The other primer pairs used in the present study have been described previously (Clair et al., 2010). Genomic DNA contamination was tested in a control reaction that did not contain reverse transcriptase. The efficiency of the primer pair was tested by amplifying subgenomic DNA.

## Results

### Discovery of a novel *ent* family gene, *entD*, in *B. cereus* ATCC 14579

**Figure 1:**
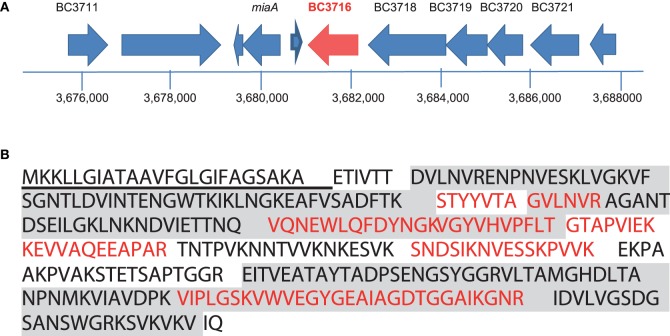
**Identification of the ***entD*** gene, based on peptide mapping to the BC_3716 locus**. **(A)** Genetic organization of the BC_3716 chromosomal region of *B. cereus* ATCC 14579. Large arrows represent the open reading frame identified in strain 14579, and their orientation shows the transcriptional direction. Only the names of ORF-encoding proteins with predicted metabolic functions are indicated. **(B)** Amino acid sequence of the *entD* product. Peptides detected by nanoLC-MS/MS are in red and boldfaced. The signal peptide is underlined. The SH3 domains located at the N terminus and the 3D domain located at the C terminus are colored in gray.

Proteogenomic analysis of the *B. cereus* ATCC 14579 exoproteome identified 4 peptides that could be mapped to the BC_3716 locus (Figure 1A), which was annotated as a noncoding pseudogene (Ivanova et al., 2003). Protein BLAST analysis of the identified peptides resulted in 100% identity matches to proteins annotated as putative enterotoxin/cell wall binding proteins or hypothetical proteins in other *B. cereus* group strains, suggesting a DNA sequencing error. We sequenced a 1429 bp PCR product encompassing BC_3716 and identified a frameshift error due to the insertion of a cytosine at the genomic position 3681749 of the annotated NC_004722 chromosome (Figure S1). The corrected sequence of the *B. cereus* ATCC 14579 BC_3716 open reading frame comprised 987 bp and was deposited in the NCBI database (BankIt1833097 Bacillus KT159458).

We investigated the transcription of BC_3716 in *B. cereus* ATCC 14579 cells grown aerobically in unregulated batch cultures on glucose-containing MOD medium by reverse transcription PCR analysis. The results indicated that early exponential growth phase (μ = μ_max_) supported the highest expression of this gene (Table S1). A transcriptional start site (G) located 26 bp from the translational start site (ATG) was determined by 5′PCR (Figure S1). Upstream of this transcriptional start site, we identified a putative housekeeping σA type -10 sequence (5′-TATAAT-3′) and a σD type -35 sequence (5′-CTAAA-3′). The BC_3716 locus was preceded by a putative operon BC3720-BC3718 and followed by a potential terminator loop (ΔG° = −19.8 kcal/mol), suggesting that it may be transcribed as a single unit.

The corrected BC_3716 coding sequence encodes a protein of 316 amino acids with an estimated molecular mass of 33463.9 Da and an isoelectric point of 8.93 (Figure 1B). The first 24 residues of the N-terminal sequence encoded a peptide signal, which was probably cleaved in the mature protein. The protein exhibits two N-terminus SH3_3 domains (PF08239) known to be involved in protein-protein interactions together with a C-terminus putative cell wall binding domain named 3D domain (PF06725) (http://pfam.xfam.org/search/sequence). The protein encoded by BC_3716 shares high similarities with the exoproteins EntA, EntB, and EntC (69, 63, and 65% sequence identity, respectively Figure S2) (Clair et al., 2010). Based on these significant similarities, we proposed to name the protein encoded by BC_3716, EntD, and the corresponding gene *entD*.

### Cell growth and division of Δ*entD*

To investigate the role of *entD*, we constructed a Δ*entD* mutant by introducing a spectinomycin-resistance cassette into the corresponding gene of *B. cereus* strain ATCC 14579. We did not detect *entD* mRNA by RT-PCR nor peptides assigned to EntD by proteomic analyses, proving that the genomic disruption of the gene generated a null allele. Growth features of the Δ*entD* mutant and the parental ATCC 14579 strain were compared. Table 1 shows that *entD* disruption significantly decreased the *B. cereus* growth rate and the acetate overflow (by ~50%) without a significant change in final biomass, probably by decreasing the glucose uptake rate (Rosenfeld et al., 2005). We tested whether anaerobic growth of *B. cereus* cells on glucose-containing MOD medium was altered by *entD* disruption. The results showed that the Δ*entD* mutant grew slower than the wild-type (μ_max_ = 0.50 ± 0.05 compared to 0.70 ± 0.02). These results indicate that *entD* disruption impacts glucose catabolism in an oxygen-independent manner.

**Table 1:** **Growth parameters and yield of end products obtained during pH- non-controlled batch cultures of ***B. cereus*** Δ***entD*** and its parental strain ATCC 14579^a^**.

	*****B. cereus*** strains**	
	**WT**	**Δ*entD***
ƛ (h)	2.41 ± 0.41	2.43 ± 0.54
μ_max_(h^−1^)	1.30 ± 0.19	0.69 ± 0.12
Final OD_600_	1.20 ± 0.12	1.44 ± 0.10
Final biomass (mg.L^−1^)	328 ± 23	326 ± 27
Final pH	5.36 ± 0.02	5.57 ± 0.02
Maximal specific glucose consumption (mmol.g^−1^.h^−1^)	72 ± 6	40 ± 4
Y_biomass_ (g.mol^−1^)	18 ± 6	17 ± 4
Y_acetate_(g.mol^−1^)	0.25 ± 0.05	0.13 ± 0.03

a *Cultures were grown on MOD medium containing 30 mM glucose. Values are means of results from triplicate cultures*.

**Figure 2:**
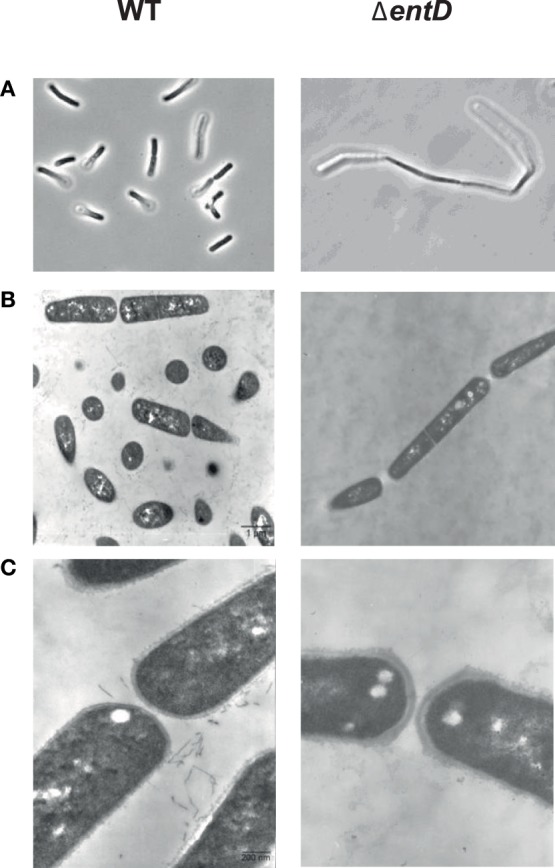
**Microscopic observation of cells in exponential growth phase cultures of ***B. cereus*** wild-type and Δ***entD*** strains**. **(A)** Phase contrast microscopy × 1000. **(B)** Thin-layer electron microscopy × 7000. **(C)** Thin-layer electron microscopy × 35,000. Cultures were grown in MOD medium supplemented with 30 mM glucose under aerobiosis. Samples were harvested during exponential growth phase. Photographs shown in the figure are of representative samples from three independently grown cultures.

The effects of *entD* disruption on cell morphology during growth were studied by phase-contrast microscopy (Figure 2A). The chains of cells of the parental strain were shortest during exponential growth phase (2 cells) and reached their maximum length (4–8 cells) as the culture entered stationary phase. The Δ*entD* mutant showed a 2- to 3-fold increase in chain length relative to wild-type during the exponential phase. To ascertain that Δ*entD* did not impair cell division, we compared the capacity of the mutant and wild-type strains to form colony forming units on LB agar plates. Neither of the assays supported a difference between the Δ*entD* and wild-type strains (data not shown). When *B. cereus* cells were viewed by thin-section transmission electron microscopy, we noted that Δ*entD* cells tended to remain attached together (Figure 2B) and the thickness of the Δ*entD* cell wall was greater than in WT cells (Figure 2C). Taken together, these results suggest that *entD* mutation disturbs the cell wall and perturbs cell separation. We then examined the consequences of cell wall disturbance on autolytic activity in exponentially growing cells. The autolytic rate was measured after 72 h at 37°C (Raddadi et al., 2005). We found that Δ*entD* had a higher autolytic rate (84 ± 3%) than wild-type (77 ± 2%).

Complementation for growth of the nonpolar *entD* mutant in *B. cereus* ATCC 14579 was not possible using a multicopy pHT304-based plasmid. To ascertain that *entD* was transcribed and translated from the plasmid, we measured the abundance level of *entD* mRNA by RT-PCR and the abundance level of EntD by MS/MS (Table S1). The results showed that *entD* was overexpressed in the complemented strain (Δ*entD*-pHT304*entD*) compared with the wild-type strain (WT/pHT304). This probably explains why the wild-type phenotype was not restored by complementation (Sprynski et al., 2012).

### Proteomic response to *entD* disruption during active growth

**Figure 3:**
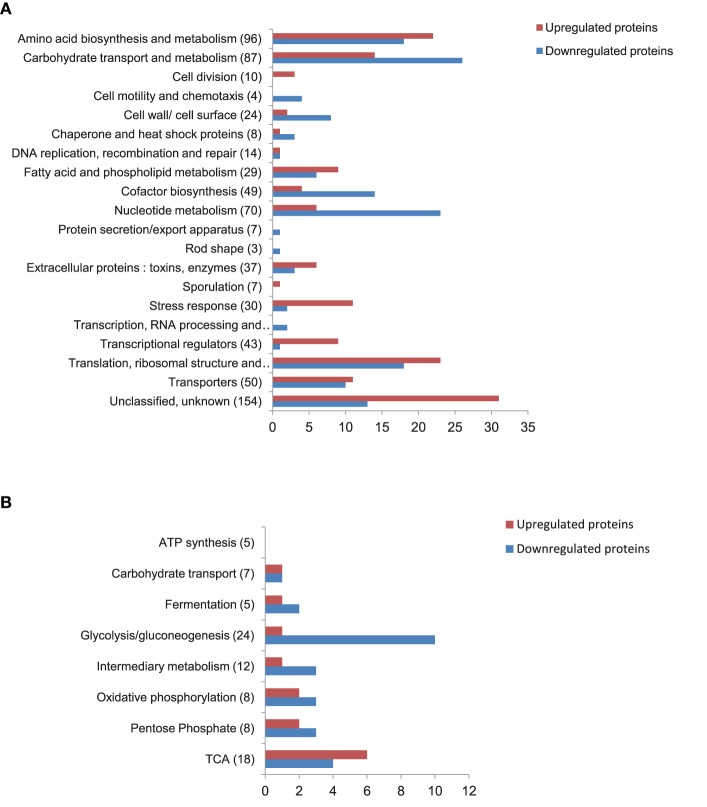
**Distribution of cellular proteins identified by shotgun proteomics showing significant abundance level changes (***p*** < 0.05) between wild-type and Δ***entD*** mutant strains**. **(A)** Distribution of downregulated (blue) and upregulated (red) proteins into 20 groups according to their functions. The number of total proteins identified in each functional group is given in brackets. **(B)** Sub distribution of downregulated (blue) and upregulated (red) proteins classified into the carbohydrate transport and central metabolism functional group. The number of total proteins identified in each of the 8 subgroups is given in brackets.

To explain the growth impairment of Δ*entD* cells, we analyzed the cellular proteome changes caused by *entD* disruption in exponentially grown cells (μ = μ_max_± 5%, Figure S3) by means of a label-free proteomic strategy conducted on triplicate biological samples for the Δ*entD* mutant and the wild-type strain (Clair et al., 2013). A total of 41,998 and 52,169 MS/MS spectra were recorded for Δ*entD* and WT, respectively (Table S2). From Δ*entD* and WT cells, 637 and 715 proteins were identified, respectively. Supplemental Table S3 presents the list of these proteins categorized into 20 functional groups. We identified 308 proteins with significant abundance level changes (*p* < 0.05) in Δ*entD* compared with WT, based on spectral counts (Table S4). Among these, 154 proteins were upregulated in the Δ*entD* mutant compared with WT, while 154 were downregulated. Figure 3A showed that Δ*entD* disruption alters most of the functional categories. However, it did not similarly alter the functional categories and subcategories in terms of the ratio of upregulated vs. downregulated proteins.

#### Focus on glucose catabolism

**Figure 4:**
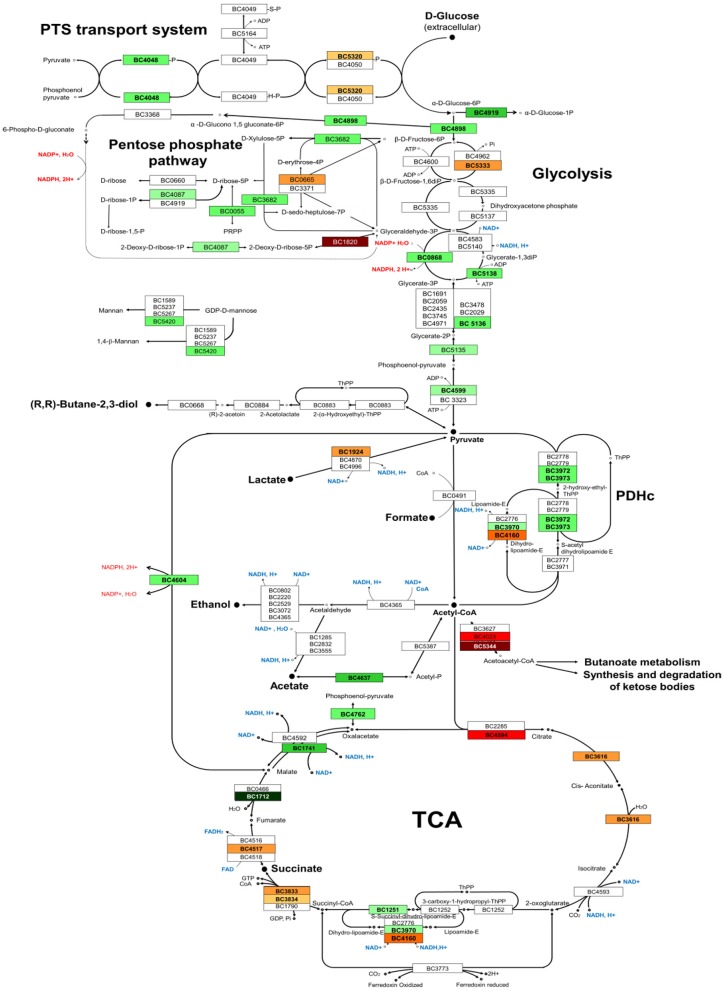
**Overview of the glucose catabolic pathways utilized by ***B. cereus*** ATCC 14579**. The proteins detected in this study are indicated by their BC numbers. Protein names and functions are listed in Table S3. The form filling indicates fold-change values that satisfied the Student's *t*-test statistical criteria (*p* < 0.05). Red and green BC numbers indicated significant increase and decrease, respectively, of abundance level of the proteins in the Δ*entD* mutant compared with wild-type.

The functional class named carbohydrate transport and central metabolism was subdivided into 8 groups according to the functional criteria: (1) ATP synthesis, (2) carbohydrate transport, (3) fermentation, (4) glycolysis/gluconeogenesis, (5) intermediary metabolism, (6) oxidative phosphorylation, (7) pentose phosphate pathway, and (8) tricarboxylic acid (TCA) cycle. Figure 3B shows that proteins related to glycolysis were mainly downregulated in the Δ*entD* mutant, which supports the lower glycolytic rate observed in this strain (Table 1). Glycolysis produces pyruvate, which is then decarboxylated into acetyl-CoA via the pyruvate dehydrogenase complex (PDC). Three components of this complex were detected in our study (BC_3970, BC_3972, and BC_3973) and all of them showed a decrease in their abundance in the mutant strain (Figure 4). The reactions catalyzed by PDC serve to interconnect the metabolic pathways of glycolysis, acetate metabolism, and fatty acid oxidation to the TCA cycle. Interestingly, the abundance levels of the two proteins that convert acetyl-CoA into acetate (phosphotransacetylase, Pta, BC_5387) and acetate kinase (Ack, BC_4637) were significantly lower in Δ*entD* (Table S4, Figure 4), accordingly to the lower production of acetate in this strain. In contrast, the quantities of acetyltransferases that convert acetyl-CoA into acetoacetylCoA were significantly increased in the mutant strain. Citrate synthase (CitZ, BC_4594), which condensates acetyl-CoA with oxaloacetate to form citrate, was significantly more abundantly detected in the mutant strain compared with the wild-type strain. In contrast, we noted a significant downregulation of fumarate dehydrogenase (Fum B, BC_1712) and malate dehydrogenase (Mdh, BC_1741), which converts malate into oxaloacetate (Table S4, Figure 4). Taken together these changes may drive the TCA cycle in the forward direction and allow high ATP generation in a background of a less active glycolysis. Consistent with these proteomic results, we did not observe growth yield change between the mutant and the WT strains (Table 1).

Five proteins assigned to the pentose phosphate pathways showed significant abundance level changes (Table S4, Figure 4). One of these, the deoxyribose-phosphate aldolase (DeoC), was strongly increased in the mutant strain (log_2_ = 6.6, *p* < 0.001). DeoC catalyzes the conversion of 2-deoxy-D-ribose-5-phosphate into glyceraldehyde-3-phosphate and acetaldehyde and allows cells to maintain a balance pool of deoxynucleosides. The gene encoding DeoC (BC_1820) belongs to the DeoR regulon of *B. cereus* (http://regprecise.lbl.gov). Proteomic analyses detected another DeoR-regulated protein (Pdp, pyrimidine-nucleoside phosphorylase encoded by BC_1822), which also showed a significant abundance level increase in the mutant strain (log_2_ = 4.8, *p* = 1.10^5^). This protein is involved in pyrimidine metabolism.

#### Nucleotide, amino acid and fatty acid metabolism, and cofactor biosynthesis

The majority of the differentially expressed enzymes involved in the biosynthesis of nucleotides were downregulated in the Δ*entD* mutant (Figure 3A). However, like Pdp, the nucleotide diphosphate kinase Ndk (BC_1515) was upregulated in the mutant strain (log_2_ = 1.3, *p* = 0.01). Ndk catalyzes the reversible transfer of the 5′-terminal phosphate from NTPs to NDPs (or their deoxy derivatives) to generate specific NTPs or dNTPs and plays an important role in bacterial growth, virulence, cell signaling and polysaccharide synthesis (Chakrabarty, 1998). Thirteen of the 49 proteins classified as cofactor biosynthesis-related proteins were downregulated in the Δ*entD* mutant. This was particularly marked for proteins involved in thiamine biosynthesis (Table S4). In contrast, 4 proteins were significantly upregulated in the Δ*entD* strain. Among these, we found the inorganic polyphosphate/ATP-NAD kinase PpnK (BC_4642, log_2_ = 2.7, *p* < 0.01), which is regarded as a key enzyme in NAPH synthesis and, hence, in numerous cellular processes (Mori et al., 2004). Interestingly, the Δ*entD* mutation favored the upregulation of proteins related to fatty acid and phospholipid metabolism and amino acid metabolism (Figure 3A).

#### Cell wall

The majority of the differentially expressed cell wall-associated proteins (9/10) were downregulated in the Δ*entD* strain compared with wild-type, including 5 proteins that were downregulated by < 2-fold (Table S4). Furthermore, 4 were proteins (BC_5264, BC_5272, BC_5277, BC_5278) that are encoded by genes belonging to the 20 kb chromosomal cluster (BC5263–BC5279), which is involved in polysaccharide biosynthesis (Ivanova et al., 2003; Hwang et al., 2014). We also noted the low *p*-value associated with the log_2_ (fold-change) of BC_5273 (log_2_ = −0.9, *p* = 0.053), indicating that it is reasonably likely that the PalI protein (Hwang et al., 2014) is less abundant in the mutant strain than in the WT strain. In addition, we noted the significant upregulation of a putative N-acetylmuramoyl-L-alanine amidase (CwlC, BC_5234, log_2_ = 2.1 *p* = 0.009). Globally, these changes may contribute to the cell wall ultrastructure difference observed between the mutant and the wild-type (Figure 2C).

#### Stress response

**Figure 5:**
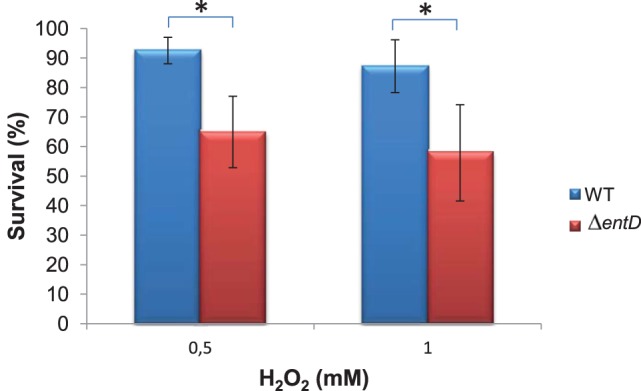
**Survival of wild-type and Δ***entD*** mutant cells toward external hydrogen peroxide insult**. Cells were grown in liquid culture to the mid-exponential growth phase, and subjected to 0.5 and 1 mM H_2_O_2_, respectively. Colony forming units per mL were counted and expressed as (N/N0) × 100. Errors bars represent the standard deviation from 6 independent measures. Significant differences (*p* < 0.05) between wild-type and mutant strains are indicated with asterisks.

Proteins classified in the stress response group were mainly upregulated by the Δ*entD* mutation (Figure 3A). In particular, the Δ*entD* mutation significantly increased the abundance level of (i) components of the general stress response system, such as the universal stress protein BC_4625 and the Ter proteins (Taylor and Zhulin, 1999; Anantharaman et al., 2012), (ii) thiol-specific stress-related proteins, such as the azoreductase (BC_2194) (Leelakriangsak et al., 2008), the thioredoxin-dependent thiol peroxidase (BC_0517), and the alkyl hydroperoxide reductase (BC_0377), and (iii) other stress-related proteins (Table S4). The higher abundance of these proteins may reflect increased endogenous ROS production in Δ*entD* growing cells. Increased endogenous ROS production can translate into increased killing by oxidants (Brynildsen et al., 2013). To evaluate the impact of EntD deficiency on *B. cereus* resistance to oxidant, *B. cereus* cells were exposed to hydrogen peroxide. Figure 5 shows that aerobically grown Δ*entD* cells were more susceptible to H_2_O_2_ deleterious effects than wild-type cells.

#### Transcriptional regulators

The transcriptional regulators ResD, Rex, and AbrB are central regulators of the primary metabolism in *B. cereus* (Duport et al., 2006; Lucking et al., 2009; Laouami et al., 2014). Their abundance level changes were significantly modified in the Δ*entD* mutant [log_2_ (fold-change) = −0.6, 0.9 and 1.2, respectively, with *p* < 0.05, Table S4]. Besides Rex and AbrB, 4 putative transcriptional regulators showed significant increases in their abundance levels in the Δ*entD* mutant (log_2_ > 2, *p* < 0.05). Among these are listed two regulators of the Ars family and one regulator of the Mar family, which control many cellular processes including oxidative stress response (Lebreton et al., 2012; Clair et al., 2013).

#### Predicted secreted protein

The cellular proteome contains proteins that are predicted to be extracellular proteins, such as flagellum components, degradation- and adhesion-related proteins and toxin-related proteins, as these are produced before being secreted. All of these proteins encompass signal peptides in their N-terminal primary structure and/or transmembrane helices. Proteins related to motility were significantly downregulated in the Δ*entD* mutant, while some of those related to degradation and adhesion appeared mostly to be upregulated (Table S4). These data suggested that EntD may be a regulator of virulence-associated extracellular proteins in *B. cereus*. To confirm this possibility, we performed a detailed analysis of the *B. cereus* exoproteome in the presence and absence of EntD.

### Insights into the exoproteome of the Δ*entD* mutant

**Figure 6:**
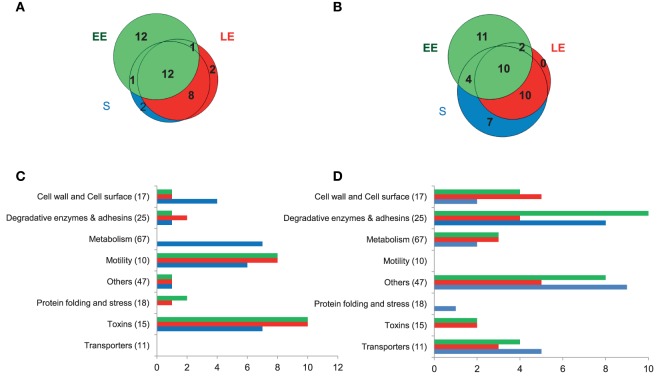
**Distribution of extracellular proteins detected by shotgun proteomics showing significant abundance level changes (***p*** < 0.05) between wild-type and Δ***entD*** mutant strains**. **(A,B)** Venn diagrams showing the numbers of downregulated **(A)** and upregulated proteins **(B)** in the Δ*entD* mutant strain in early exponential (EE), late exponential (LE), and stationary (S) growth phases. **(C,D)** Distribution of downregulated **(C)** and upregulated **(D)** proteins into 8 groups according to their functions during the EE, LE, and S growth phases. The number of total proteins identified in each functional group is given in brackets.

The regulation of exoprotein synthesis is typically growth phase dependent (Madeira et al., 2015). We thus examined the impact of Δ*entD* on the *B. cereus* exoproteome during early exponential growth phase (EE), late exponential growth phase (LE) and stationary phase (S) (Figure S3). The global dataset comprised 68,058 MS/MS spectra, from which 28,782 were assigned to peptide sequences (Table S5). A total of 210 proteins were identified based on the confident detection of at least two different peptides (Table S6). Changes in the abundance levels of proteins were analyzed according to their functional classification (Table S7). The data indicate that 82 proteins were differentially expressed in the Δ*entD* mutant compared with wild-type. The Venn diagrams presented in Figure 6 show the growth phase distribution of the 38 downregulated (Figure 6A) and 44 upregulated proteins (Figure 6B), respectively. Only 12 and 10 proteins were found to be down- or upregulated in all three growth stages, indicating that EntD modulates the *B. cereus* exoproteome mainly in a growth-phase dependent manner. Figures 6C,D show that motility- and toxin-related proteins are mainly downregulated during growth, while degradative enzymes and adhesins, transport and cell wall/cell surface–related proteins are mainly upregulated.

#### Focus on motility

**Figure 7:**
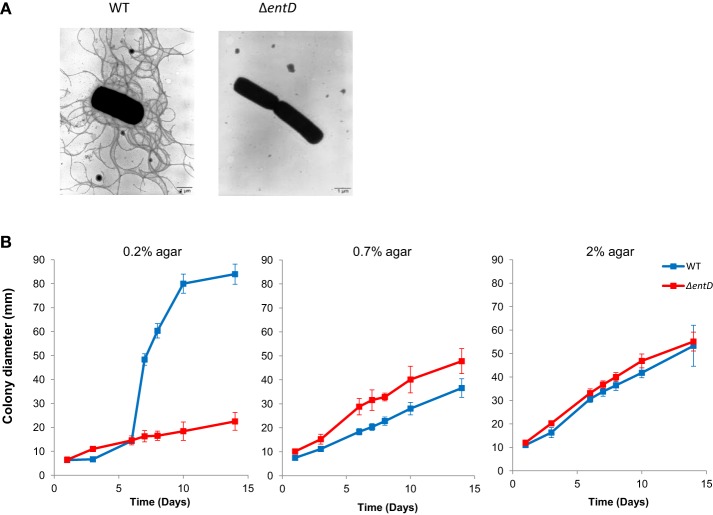
**Flagellation and motility of ***B. cereus*** wild-type and Δ***entD*** mutant strains. (A)** Negative staining electron micrographs (× 7000) of wild-type and Δ*entD* mutant strains. **(B)** Motility of wild-type and Δ*entD* mutant strains. Diameters of motility haloes were measured during 2 weeks on TrB agar plates containing 0.2, 0.7, and 2% agar, respectively. The data shown are the mean ± standard deviations of triplicates. Statistical differences between WT and mutant strains were evaluated with the Student's *t*-test.

Both the cellular proteome and exoproteome showed lower accumulation of flagellum components in the Δ*entD* mutant compared with wild-type (Tables S4, S7). Accordingly, electron microscopy revealed that Δ*entD* cells were non-flagellated while the wild-type cells exhibited a peritrichous distribution of flagella (Figure 7A). The absence of flagellation observed in Δ*entD* cells prompted us to evaluate whether these cells displayed altered motility. Analysis of swimming motility was performed by measuring the diameters of growth haloes on TrB solidified with 0.2% agar. As shown in Figure 7B, swimming was strongly reduced for Δ*entD* strain cells (23 ± 4 mm) when compared with the wild-type cells (83 ± 1 mm) after 14 days. Mutant cells were also inoculated on TrB solidified with 0.7% agar for swarming and under the same growth conditions except for a harder surface (2% agar) as a non-swarming control (Salvetti et al., 2011). Figure 7B indicated that Δ*entD* mutant cells exhibited higher mobility than wild-type cells on 0.7% agar TrB. Taken together, these results indicated that EntD deficiency alters *B. cereus* motility. Motility and biofilm formation are related differentiation processes in Gram-positive bacteria (Abee et al., 2011). We thus investigated the impact of the Δ*entD* mutation on the capacity of *B. cereus* to form a biofilm. Biofilm formation by the Δ*entD* mutant (OD_590_ = 1.11 ± 0.28) was about two-fold higher that biofilm formation by the wild-type (OD_590_ = 0.60 ± 0.17). Therefore, the Δ*entD* mutation appeared to have a positive role in biofilm formation.

#### Focus on cell wall-associated enzymes and degradative enzymes

Exoproteome analysis confirmed the impact of EntD deficiency on the distribution pattern of cell wall-associated enzymes and showed that this impact is growth phase dependent (Figure 6, Table S7). EntD deficiency also impacted the abundance level of degradative enzymes in a growth phase-dependent manner. Only 4 proteins showed abundance level changes in the 3 growth phases: the InhA2 metalloprotease (BC_0666) and the phosphatidylinositol-specific phospholipase C enzyme (PlcA, BC_3761), which were more abundant in the Δ*entD* exoproteome than in the wild-type, and the collagenase ColC (BC_0556), which was less abundant. These proteins are recognized as virulence factors (Fedhila et al., 2002; Karunakaran and Biggs, 2011).

#### Focus on toxin production

**Figure 8:**
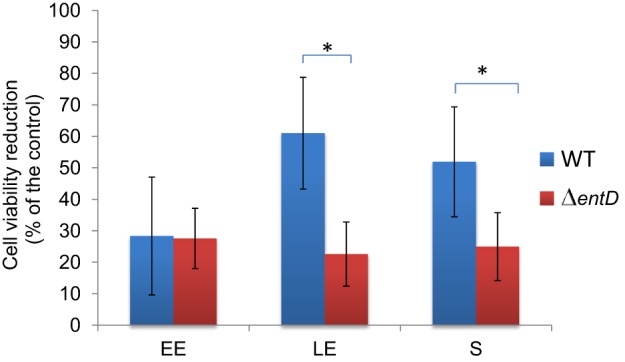
**Evaluation of cytotoxicity using MTT bioassay**. Cytotoxicity of *B. cereus* wild-type and Δ*entD* mutant supernatants was assessed on epithelium-like differentiated Caco-2 cells. Control cells were incubated with MOD medium. The percentage of cell viability reduction (MTT-reduction) was the mean of at least 4 independent treatments, and was related to control cells. The statistical difference between wild-type and Δ*entD* strains was evaluated with the Student's *t*-test.^*^ = *p* < 0.05.

EntB was not detected in the Δ*entD* culture filtrate (Table S6). In contrast, the abundance levels of EntA and EntC were significantly increased in the LE and S exoproteomes of Δ*entD* cells (log_2_> 0.8, *p* < 0.05, Table S7). This suggests that the lack of EntD and EntB may be partially offset by EntA and EntC. Like EntB, none of the 3 Nhe components was detected in the Δ*entD* exoproteome (Table S6). The abundance levels of CytK and the 3 Hbl components were significantly decreased in the Δ*entD* exoproteome, especially during the LE and S growth phases (log_2_ < −0.6, *p* < 0.05, Table S7). RT-PCR experiments showed that the mRNA level of *nhe* was strongly decreased compared to *cytK* and *hbl* (log_2_ = −16 compared to −0.5, *p* < 0.05) in the mutant strain compared with the wild-type strain. These results thus are in agreement with the proteomic data. Gastrointestinal (GI) toxicity of *B. cereus*, mainly caused by the cytotoxins Nhe, Hbl, and CytK, was evaluated on differentiated Caco-2 cells (mimicking the GI epithelium) using the MTT cell viability/metabolic assay (Hidalgo et al., 1989; Vidau et al., 2009). Figure 8 shows the cytotoxic effects of EE, LE, and S filtrate supernatants prepared from Δ*entD* and wild-type cells after 24 h contact with Caco-2 cells when incubated at 100 μg proteins. We noted that Caco-2 cells were more sensitive to wild-type (more than 50% cell viability reduction with LE and S filtrate supernatants) than to Δ*entD* filtrate supernatant (less than 30% in all cases). We thus assume that EntD regulates the cytotoxic potential of *B. cereus* cells by modulating its exoproteome.

## Discussion

Discovering virulence factors is important in understanding bacterial pathogenesis and their interactions with the host. Proteomics is today a popular tool to discover virulence factors in pathogenic bacteria and has the advantage of defining proteins that are differentially produced, and differentially located or secreted outside of the cell (i.e., to the media), namely the exoproteome (Armengaud et al., 2012). By identifying proteins that were missed during annotation, proteogenomics is also an important tool to discover new virulence factors (Armengaud, 2010; Renuse et al., 2011). A proteogenomic study allowed us to identify EntD, which shares common domains with the previously identified exoproteins EntA, EntB, and EntC (Clair et al., 2010). Nothing is known about the function of EntD. Therefore, we made a knockout mutant and performed a comparative proteomic analysis using high-throughput nanoLC-MS/MS with a high-resolution Orbitrap mass spectrometer.

The abundance level of a large number of proteins involved in central metabolism was significantly altered in the Δ*entD* mutant. This leads to catabolic readjustments and a lower growth rate in nutrient-limited conditions. The differences in catabolic activities might reflect a higher anabolic, and thus energy, requirement in the Δ*entD* mutant (Koebmann et al., 2002). Dysregulation of central metabolism could affect the NADH/NAD^+^ ratio in the cell, which is likely to perturb the oxidative stress response. This was corroborated by significant level changes of oxidative stress related proteins and the highest sensitivity of Δ*entD* cells toward external antioxidants. However, we cannot exclude a direct link between the increased sensitivity of the Δ*entD* mutant to external oxidant and the modification of the cell wall structure.

EntD deficiency decreased the production of flagellum components and altered the distribution of cell wall-associated proteins. As a result, Δ*entD* mutant cells were non-flagellated and showed a higher capacity to stick their cell walls together. This could facilitate colony expansion during sliding motility on solid surfaces (0.7% agar plates). Indeed, unlike swarming, sliding motility does not require flagella (Henrichsen, 1972; van Gestel et al., 2015). Flagellum-dependent motility is important for biofilm formation at the air-liquid interface in microtiter plates (Houry et al., 2010). However, the Δ*entD* mutant showed a higher capacity to form a biofilm than the wild-type. We observed that the Δ*entD* mutant had a decreased sedimentation rate compared to its parental strain (data not shown). This could explain why Δ*entD* cells produce more biofilm than wild-type cells in static conditions. In conclusion, EntD plays a role in both motility and biofilm formation by regulating (i) the anchor of flagella at the cell surface throughout its synthesis, and (ii) the composition of the cell wall, as deciphered by proteomics.

EntD deficiency altered the abundance level of a range of extracellular *B. cereus* virulence factors including the PlcR-regulated Hbl, Nhe, and CytK enterotoxins (Gohar et al., 2008; Stenfors Arnesen et al., 2008). These enterotoxins are downregulated in Δ*entD* mutant supernatant, while the abundance level of their specific regulator, PlcR, was unchanged. Specifically, Nhe was undetected in both the cellular and extracellular proteomes of the Δ*entD* mutant whatever the growth phase, while the Hbl abundance level was significantly decreased at the end of growth. Hbl is the major contributor to *B. cereus* cytotoxicity toward Caco-2 cells (Koehler, 2002). Our experiments showed that EntD deficiency decreased the cytotoxic activity of *B. cereus* supernatant toward Caco-2 cells only when harvested at the end of growth. This indicates that the cytotoxicity defect of the Δ*entD* mutant is mainly due to the abundance level decrease of Hbl in the *B. cereus* exoproteome.

Cellular proteome analysis showed that *entD* disruption affected the abundance level of several global transcriptional regulators. In addition, exoproteome analysis showed a growth phase-associated regulation of exoproteins in the *entD* mutant. Taken together these data indicate that some of the differences associated with the *entD* mutation are likely to be due to perturbation of other regulatory circuits. For example, proteins associated with glucose catabolism, oxidative stress, motility, and toxinogenesis are known to be regulated by transition state regulator AbrB-, NADH/NAD^+^-sensing regulator Rex-, and redox-sensing regulator ResD –dependent regulatory circuits (Hamon et al., 2004; Duport et al., 2006; Laouami et al., 2014; Lozano Goné et al., 2014). Control of motility and biofilm formation may also be dependent on abundance level changes of the putative transcriptional regulator, BC_5278, which belong to the EPS-related genome region, and the RNA chaperone Hfq (BC_3716) (Möller et al., 2014; Panda et al., 2015).

Finally, our data showed that EntD coordinates multiple cellular responses in *B. cereus* cells and modulates global regulatory circuits. This raises the question of the original native function of EntD. In wild-type cells, EntD was detected exclusively in the *B. cereus* exoproteome, according to the presence of an N-terminal secretion signal. EntD contains a positively charged 3D domain together with SH3 domains known to be involved in protein-protein interactions (Schneewind et al., 1995; Marino et al., 2002). Once translocated across the cell wall, EntD may thus remain associated with the cell wall and assemble into macromolecular structures on the cell surface. Among the possible interacting partners of EntD are its homologs EntA, EntB, and EntC (Clair et al., 2010), which showed a strongly modified distribution pattern in Δ*entD* cells. Our experimental data revealed that EntD deficiency alters the morphology and properties of the *B. cereus* cell wall. We thus propose that EntD is a protein that plays a crucial role in maintaining cell wall structure. Because cell wall structural integrity is essential for survival, we suggest that the lack of EntD leads to the activation of a cellular response that allows *B. cereus* to adapt and survive. This adaptive response may be mediated by a cell wall integrity (CWI)–like pathway (Jendretzki et al., 2011). Nucleoside diphosphate kinase (NdK) may act as a key intracellular component of this CWI pathway (Chakrabarty, 1998; Kim et al., 2014). The cellular response is the activation of a transcriptional adaptation program related to cell wall remodeling, metabolism, and virulence involving the regulators ResD (which interacts with Ndk in two-hybrid assays, unpublished data), Rex and AbrB. The final consequence of this EntD-dependent response is the modulation of growth at the benefit of the reorganization of the *B. cereus* surface (with special focus on cell surface-associated proteins) for biologically important processes, such as sliding motility and the establishment of biofilms (Garg et al., 2015).

In summary, this study shows that *B. cereus* has developed a complex regulatory network that links cell wall structure, cell growth, motility, and enterotoxin production, and coordinates surface-associated proteins. Besides EntD, this network may involve EntA, EntB, and EntC. These proteins in *B. cereus* are nearly identical to those in the very closely related pathogen *Bacillus anthracis*. *B. anthracis* is non-motile, and does not readily form biofilms and enterotoxins. However, it produces several virulence factors, also found in *B. cereus* (Pflughoeft et al., 2014). The Ent A, B, C, and D proteins may thus provide attractive targets to study both *B. cereus* and *B. anthracis* pathogenesis.

### Conflict of interest statement

The authors declare that the research was conducted in the absence of any commercial or financial relationships that could be construed as a potential conflict of interest.
