# Supplementary material for: Proteomics identifies Bacillus cereus EntD as a pivotal protein for the production of numerous virulence factors
Source: Front Microbiol. 2015 Oct 7;6:1004. doi: 10.3389/fmicb.2015.01004 (PMC4595770; doi:10.3389/fmicb.2015.01004)
Supplement: Supplementary file 1 [file Table1.DOCX]

Table S1: Changes in *entD* mRNA levels induced by growth phase, Δ*entD* mutation and *entD* complementation in trans.

|  | Log_2_(fold-change)^a^ | | |
| --- | --- | --- | --- |
|  | EE | LE | S |
| Wild-type | 0 | -1.5 | -4.3 |
| Δ*entD* | nd^b^ | nd | nd |
| Δ*entD*/pHT*entD* | +2.3 |  | -1 |

^a^ each log_2_(fold-change) represents the mean level of mRNA in the samples relative to the mean level in the EE wild-type sample. The mean values were obtained from two measurements done on triplicate independent cultures. Plus and minus indicate increased and decreased abundance levels, respectively. All log_2_ ratios expressed at above 1.0 (absolute value) are significant according to the precision of the method.

^b^ not detected
