# Supplementary figures and images for: Proteomics identifies Bacillus cereus EntD as a pivotal protein for the production of numerous virulence factors
Source: Front Microbiol. 2015 Oct 7;6:1004. doi: 10.3389/fmicb.2015.01004 (PMC4595770; doi:10.3389/fmicb.2015.01004)

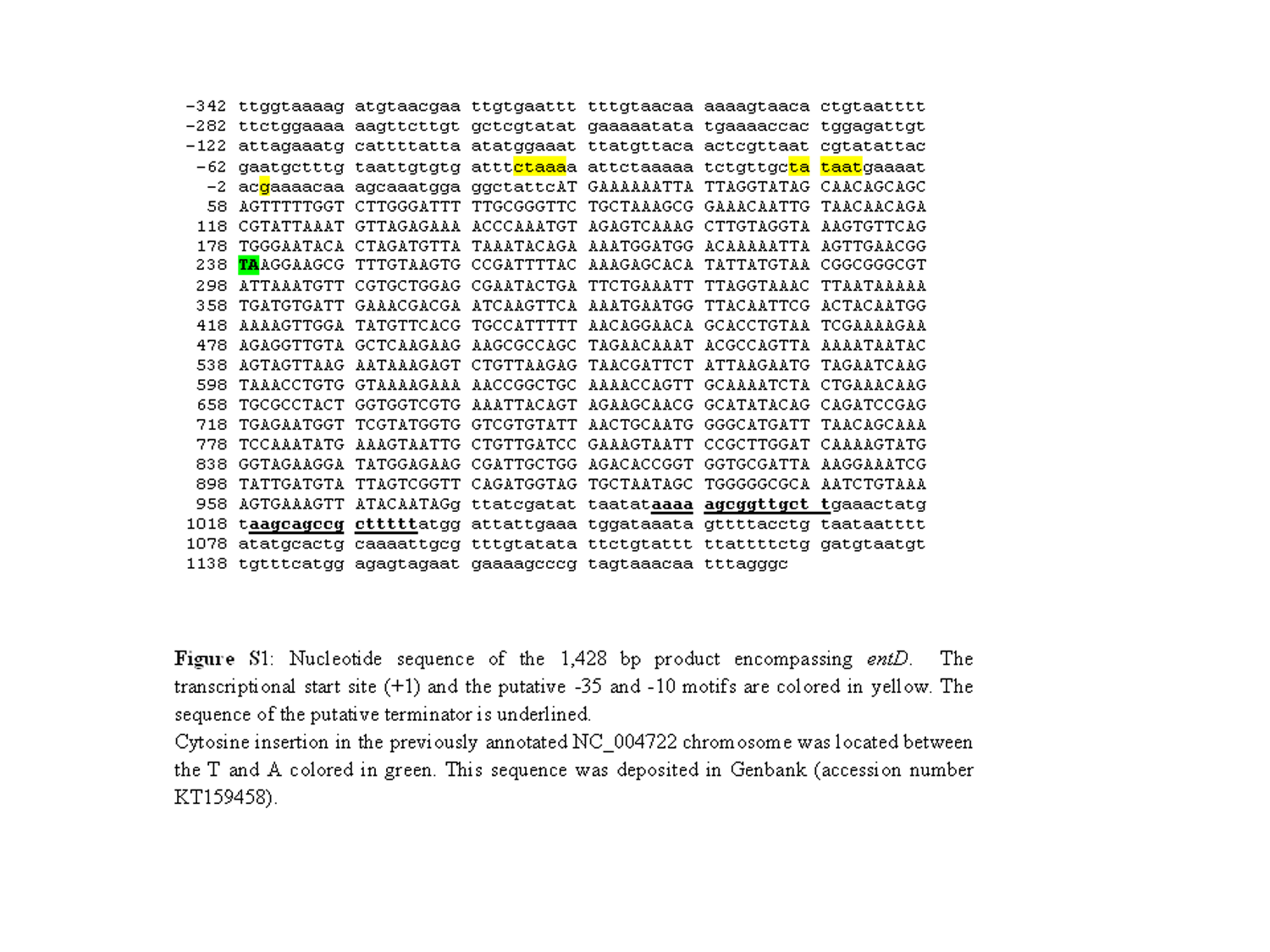

Supplement: Supplementary file 8 [file Image1.TIF]

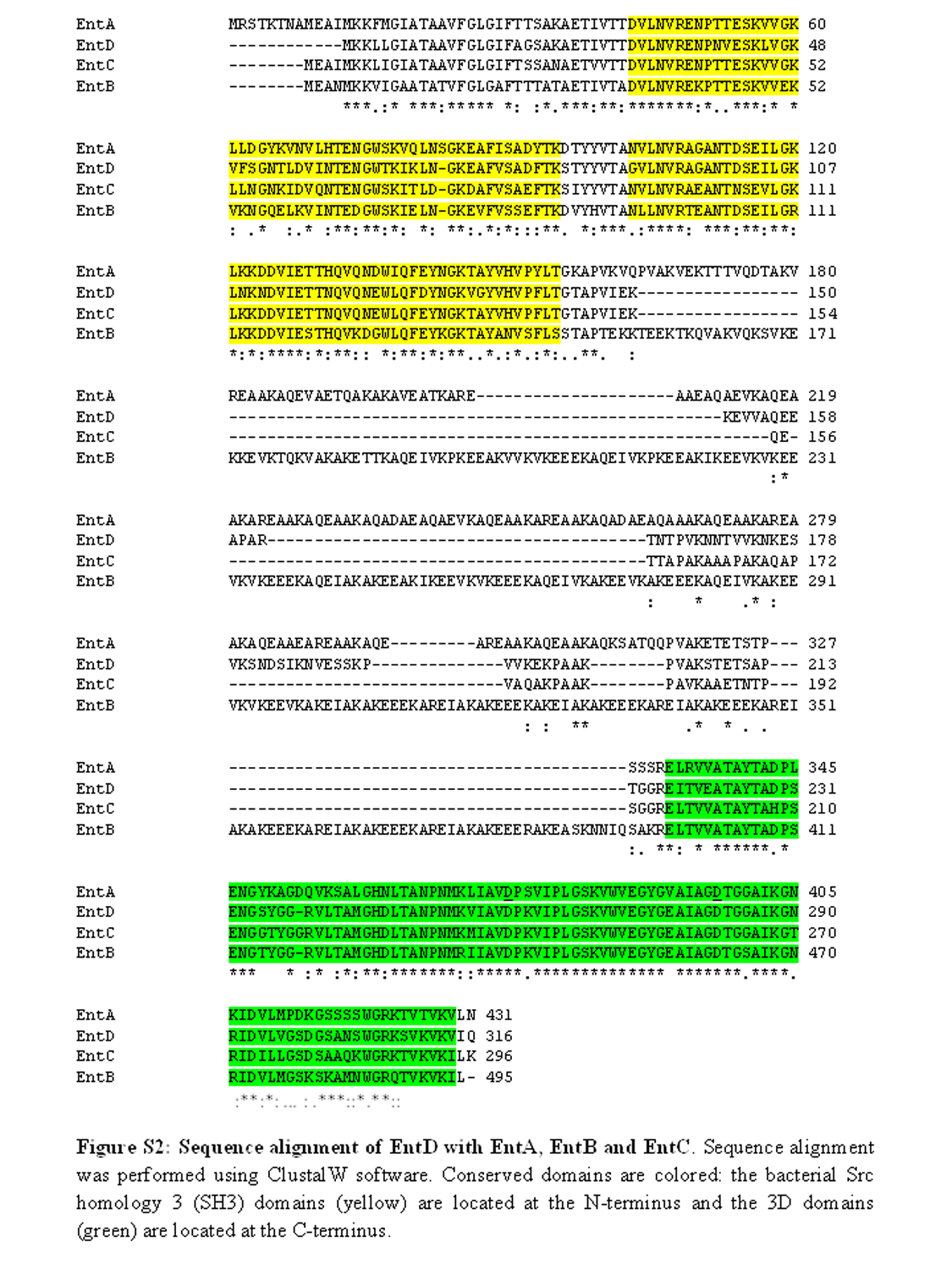

Supplement: Supplementary file 9 [file Image2.TIF]

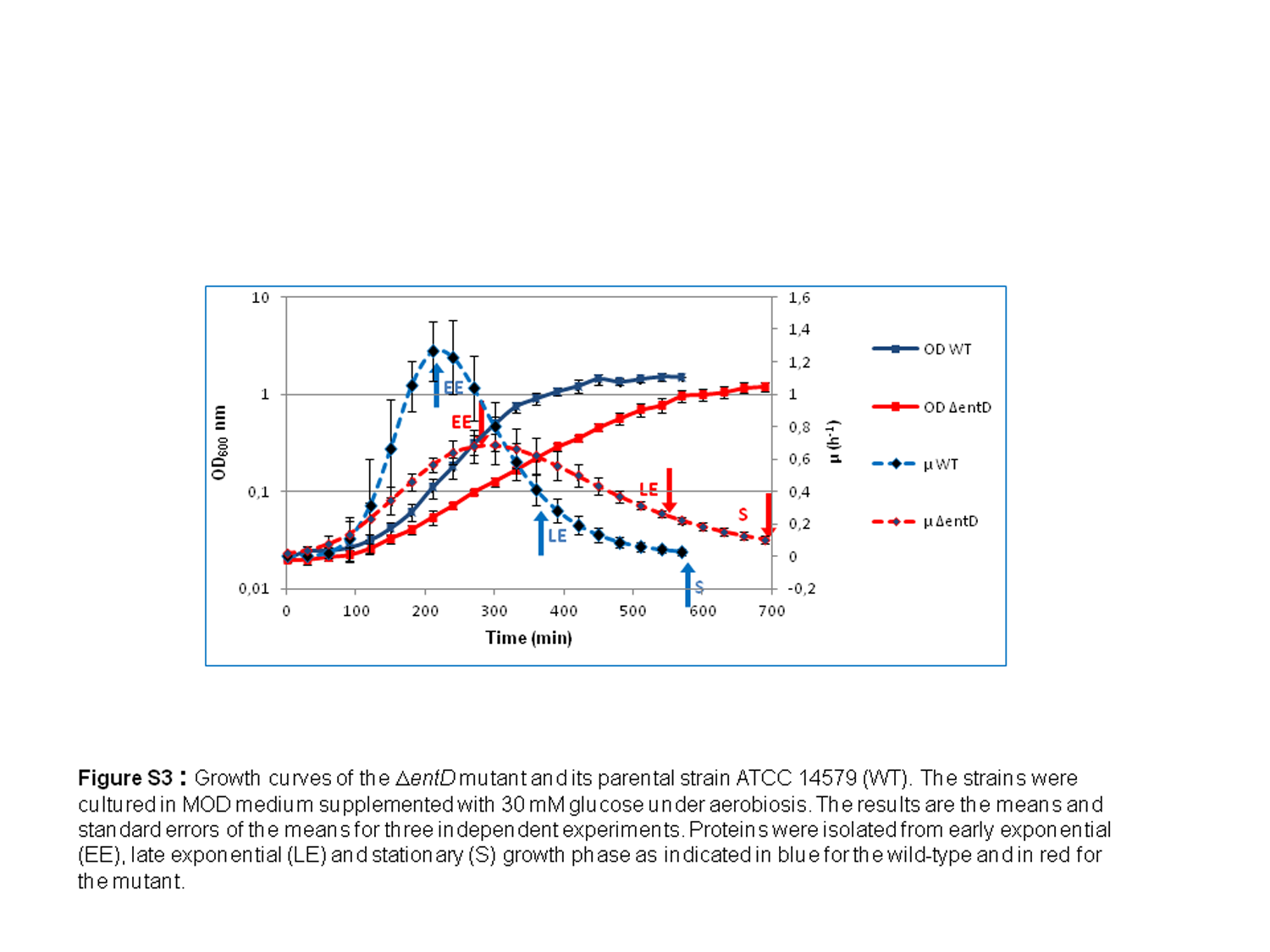

Supplement: Supplementary file 10 [file Image3.TIF]
